# Preparation and Characterization of New Inclusion Compounds Using Stable Nitroxide Radicals and an Organic 1-D Nanochannel as a Template

**DOI:** 10.3390/ma3063625

**Published:** 2010-06-09

**Authors:** Hirokazu Kobayashi, Tetsuo Asaji, Atsushi Tani

**Affiliations:** 1 Department of Chemistry, College of Humanities and Sciences, Nihon University / 3-25-40, Sakura-jo-sui, Segataya-ku, Tokyo, 156-8550, Japan; E-Mail: asaji@ chs.nihon-u.ac.jp (T.A); 2 Graduate School of Science, Osaka University / 1-1, Machikaneyama-cho, Toyonaka, Osaka, 560-0043, Japan; E-Mail: atani@ess.sci.osaka-u.ac.jp (A.T)

**Keywords:** TPP, ESR, inclusion compound, organic radical, 1-D spin chain, organic magnet

## Abstract

A new inclusion compound (IC) using di-*t*-buthyl nitroxide (DBNO) radical and tris(*o*-phenylenedioxy)cyclotriphosphazene (TPP) (**1**), which has an organic one-dimensional (1-D) nanochannel in the crystal, is reported. According to the characterization using thermogravimetric analysis (TG), ESR measurements, *etc.*, the composition of the inclusion compound was assigned as TPP:DBNO = 1:0.62. The narrowing of the isotropic ESR adsorption line of **1** was observed with a temperature increase from 103 K to room temperature. The line shape indicated a type of 1-D spin diffusion as observed in our previous study of the IC using TPP and 2,2,6,6-tetramethyl-1-piperidinyloxyl (TEMPO).

## 1. Introduction

**Scheme 1:**
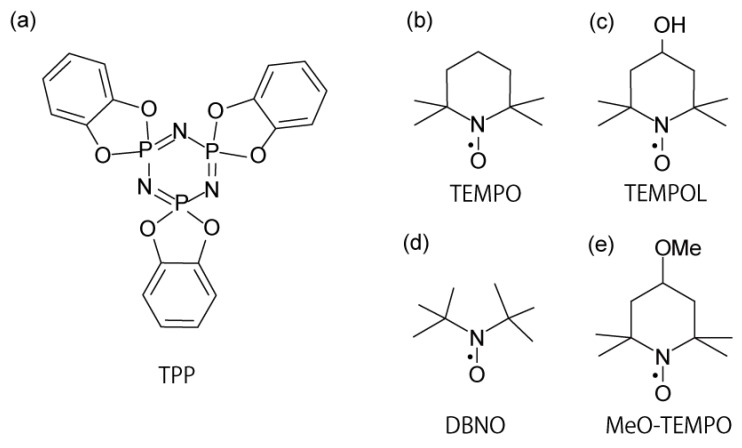
Chemical structures of the host and related guest compounds.

The crystal of tris(*o*-phenylenedioxy)cyclotriphosphazene (TPP, Scheme 1 a) [1,2,3,4] has a one-dimensional (1-D) homogeneous nanochannel [5,6,7]. In recrystallization of TPP, solvent molecules are included in the nanochannel. As the guest molecules are desorbed when heated under low pressure conditions, the structure of the 1-D nanochannel is retained and guest-free TPP enables the adsorbption of other molecules in the nanochannel; guest molecules are aligned in the TPP nanochannel in a 1-D manner. Thus, TPP can be used as a template for the 1-D arrangement of various molecules, in particular, of functional molecules, e.g., electron donors such as as I_2_ [8], dye molecules [9,10], and organic radicals [11,12]. Therefore, TPP is an attractive and remarkable compound from the point of view of the design and development of a new functional compound with developed anisotropic physical properties.

The pore diameter of TPP is adjustable from 0.46 (for guest-free TPP [13]) to 1 nm, depending on the size of guest molecules. The basic structure of the wall of the TPP nanochannel is formed by three phenyl rings of the neighboring TPP molecules in which the ring plane is parallel to the channel axis. Therefore, the wall of TPP nanochannels is *π*-electron-rich. Moreover, TPP nanochannels have irregularities when they are examined in more detail. The bottleneck gives the minimum diameter, which corresponds to the diameter of a circle close to the plane of the phenyl rings, whereas the maximum diameter corresponds to the interspace of the TPP layers. It is known that weak interactions between the phenyl rings forming the wall of the TPP nanochannels and guest molecules exist, according to the results of NMR measurements [14,15]. In the TPP nanochannels, guest molecules are uni-axially rotating around the channel axis in most cases. The molecular dynamics of guest molecules are mainly investigated using NMR, because the fast uni-axial rotation or the structural disorder of guest molecules makes the determination of the molecular structure or orientation in the nanochannels by single crystal X-ray diffraction difficult. This is even the case for TPP/benzene IC, in which the shape of guest molecules is simple and highly symmetric [16,17,18,19,20]. In addition, TPP is also a medium for gas storage because the strong affinity of the wall of the nanochannels makes it possible to include even gas molecules [21,22,23].

Recently, we developed a new inclusion compound (IC) with 1-D molecular arrangement using TPP and 2,2,6,6-tetramethyl-1-piperidinyloxyl (TEMPO, Scheme 1 b), named TPP/TEMPO IC) [24,25]. The guest-free TPP crystal can accommodate one TEMPO molecule per unit cell formed by two TPP molecules. Since TPP/TEMPO IC is constructed only of the organic host and guest molecules, it is attractive as a candidate for a new organic magnet with 1-D spin chains consisting of an unpaired electron localized on the 2p orbitals. According to the ESR measurements of TPP/TEMPO IC, a temperature-dependent 1-D inter-spin interaction between the electron spins aligned in a 1-D manner was confirmed. Since the temperature-dependent 1-D inter-spin interaction is closely related to the molecular orientation and dynamics of TEMPO in the TPP nanochannels, the temperature dependence of the ESR spectrum of the IC was investigated in detail using TPP and TEMPO diluted by diamagnetic molecules [26]. The molecular orientation and dynamics of TEMPO in the TPP nanochannels were analyzed on the basis of the ESR measurements according to the method of Freed [27]; the nitroxide groups of TEMPO molecules are oriented perpendicular to the channel axis of the TPP nanochannels, and TEMPO molecules uni-axially rotate around the axis.

For clarification of the relation between the temperature-dependent 1-D inter-spin interaction of TPP/TEMPO IC and the molecular dynamics of TEMPO in the TPP nanochannels, a new IC using a differently sized nitroxide radical to TEMPO was prepared. 4-hydroxy-2,2,6,6-tetramethyl-1-piperidinyloxyl (TEMPOL, Scheme 1 c) [28,29,30,31] was used. The preparation of the ICs using TPP and TEMPOL were attempted by gas adsorption or recrystallization in our previous study [32]. On the basis of the ESR measurements of the prepared ICs, it was found that the molecular orientation of TEMPOL in the TPP nanochannels is similar to observed in the case of TPP/TEMPO IC. Moreover, the existence of 1-D inter-spin interaction between the electron spins of TEMPOL molecules in the nanochannels was implied. However, it was hard to prepare our objective IC, *i.e.*, TPP/TEMPOL IC. This was due to the hindrance of the diffusion of TEMPOL molecules in the TPP nanochannels (in the gas adsorption method) or because of the co-inclusion of solvent molecules (in the recrystallization method). These results indicate that the preparation of “TPP/TEMPOL IC,” in which 1-D TEMPOL molecular chains are formed in the TPP nanochannels, and in which no solvent molecule is co-included, is not as easy as the preparation of TPP/TEMPO IC.

On the other hand, a smaller nitroxide radical than TEMPO, such as the di-*t*-butyl nitroxide radical (DBNO, Scheme 1 d), is more promising as a guest compound for the clarification of the relation between the inter-spin interaction and the molecular dynamics of the guest organic radicals. The principal values of the ***g*** and ***A*** tensors of DBNO were investigated in tetrametyl-1,3-cyclobutanedione at room temperature as follows: *g*_xx_ = 2.0088, *g*_yy_ = 2.0062, *g*_zz_ = 2.0022 and *A*_xx_ = 0.76 mT, *A*_yy_ = 0.60 mT, *A*_xx_ = 3.18 mT [33,34]. So far, the IC using DBNO and thiourea crystal with 1-D cavities, whose diameter is 0.7 nm, was recrystallized from methanol [35]. On the basis of the powder ESR measurements, it was found that the ***g*** and ***A*** tensors of the thiourea/DBNO IC were axial symmetric. These results indicated that the nitroxide group of DBNO is orientated perpendicular to the axis of the thiourea cavities, and that DBNO molecules are in a uni-axially fast rotating state around the cavity axis. In the TPP nanochannels, DBNO molecules, whose molecular size (0.53 × 0.70 × 0.45 nm^3^) is horizontally-long with respect to the nitroxide group, are expected to be in similar molecular orientation as in the thiourea cavity, or as that of TEMPO molecules in the TPP nanochannels. This is because the forms of the TPP nanochannels and the cavities of thiourea are similar. Actually, it is known that smaller size molecules are preferably included in the TPP nanochannels within mixed solvents, and that guest molecules are oriented in the TPP nanochannels such that the longer molecular axis is pointing parallel to the channel axis [2]. Because of the smaller molecular size of DBNO (in comparison with that of TEMPO derivatives), DBNO is anticipated to (1) pack more tightly within the TPP nanochannels, thus causing a greater dipolar broadening of ESR line, and (2) perform faster molecular re-orientational motion in the TPP nanochannels and to give greater influence on the ESR line narrowing (in comparison with the case of TPP/TEMPO IC). Since the ESR spectrum of isolated DBNO molecules was simply observed in thiourea/DBNO IC, it is interesting to observe whether the inter-spin interaction is observed or not in the ESR measurements of the IC using TPP and DBNO.

In this article, we report the preparation procedure of the IC using DBNO and TPP (compound **1**). Mesitylene was used as a solvent because it has a larger molecular size than DBNO. The characterization of the specimens was performed using thermogravimetric analysis (TG), powder X-ray diffraction (XRD), chemical analysis (CA), and ESR measurements. In particular, the temperature dependence of the line width and shape of the ESR spectrum were investigated in detail. In addition, the dimensionality of the inter-spin interaction of compound **1** was discussed on the basis of the ESR measurements. On the other hand, for the preparations using larger nitroxide radicals than TEMPO, we reattempted the preparation of the IC using TPP and TEMPOL (compound **2**), *i.e.*, TPP/TEMPOL IC, using a different procedure than in our previous study [32]; recrystallization at the lowest possible temperature. In this case, close attention was paid to the reaction temperature to prevent thermal decomposition of TEMPOL by heating. In addition, the preparation of the IC using TPP and 4-methoxy-2,2,6,6-tetramethyl-1-piperidinyloxyl (MeO-TEMPO, Scheme 1 e) was attempted by gas adsorption or recrystallization. However, this was difficult for several reasons (see below). These results provide important ideas for the design of a new organic magnet.

## 2. Results and Discussion

### 2.1. Sample characterization of compound **1**

**Figure 1:**
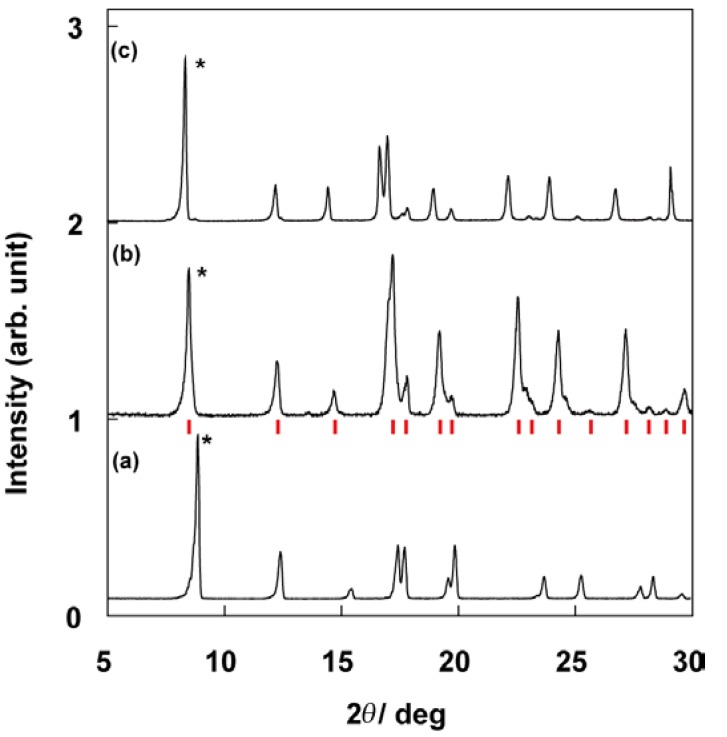
Powder X-ray pattern of guest-free TPP **(a)**, compound **1 (b)**, and TPP/TEMPO IC **(c)** [24]. The reflection patterns are similar to one another. Each peak in (b) is totally shifted to the lower degree side as compared with (a), and the shift is not as much as (c) (e.g., see the peaks corresponding to (100) plane of guest-free TPP marked by asterisks). The red bars under **(b)** show the peak positions calculated as compound **1** belonging to the same space group as **(a)**.

Sample characterization of compound **1** was performed using CA, TG, XRD, and ESR spin concentration measurements. The CA of **1** revealed the following: H, 3.99%; C, 50.55%; N, 8.96%. These results did not coincide with the composition: H, 3.98%; C, 49.73%; N, 9.22% of the expected compound with TPP:DBNO = 1:0.5 in contrast with the case of TPP/TEMPO IC, which has the composition of TPP:TEMPO = 1:0.5 within an experimental error of ± 0.3 % (see 1. Introduction). The results closely approximated the composition of TPP:DBNO = 1:0.62 within experimental error: H, 4.26%; C, 50.27%; N, 9.24%. The CA results imply that more than one DBNO molecule is accommodated per TPP unit cell formed by two TPP molecules. The results of the TG are consistent with those of the CA. The weight ratio of the desorbed compounds from **1** was 15.6%, and this coincides well with the calculation in the case of TPP:DBNO = 1:0.62, *i.e.*, 15.9%. Again, the calculation in the case of TPP:DBNO = 1:0.5, 13.6%, is slightly smaller than the experimental results. In addition, the results of the ESR spin concentration of **1**, 6.4 × 10^20^ g^-1^, was coincident with the estimation in TPP:DBNO = 1:0.62, 6.8 × 10^20^ g^-1^, whereas in TPP:DBNO = 1:0.5, the estimation is at most 5.7 × 10^20^ g^-1^. Figure 1 shows the powder X-ray pattern of guest-free TPP (a), compound **1** (b), and TPP/TEMPO IC (c) [24]. Those reflection patterns are arranged in order of size of guest compounds (*i.e.*., (a) < (b) < (c)). They are similar to one another.  Each peak in Figure 1(b) is totally shifted to the lower degree side as compared with Figure 1(a), but the shift is not as large as Figure 1(c). (e.g., see the peaks corresponding to (100) plane of guest-free TPP marked by asterisks). This result implies that 1), in **1**, the similar structure of the 1-D TPP nanochannels to the guest-free TPP is retained, 2) the inclusion of DBNO molecules in the nanochannels is responsible for the peak shift, *i.e.*., the expansion of the diameter of the TPP nanochannels, and 3) the diameter of the TPP nanochannel in **1** is not expanded as much as TPP/TEMPO IC. According to the results of various characterizations, compound **1** was assigned to be TPP/(DBNO)_0.62_ IC.

The space group and the cell parameters of guest-free TPP are known as P6_3_/m, and *a* = 1.1454 nm and *c* = 1.0160 nm, respectively [13]. The expanded cell parameters were calculated as *a* = 1.202 nm and *c* = 0.998 nm. This assumes that the TPP nanochannel moiety of **1** belongs to the same space group as that of guest-free TPP. The peak positions based on the calculation were consistent with the experimental results, as shown by the red bars under (b).

The minimum and maximum channel diameters of the TPP nanochannels of **1** (see 1. Introduction) were calculated to be 0.50 ± 0.02 and 0.76 ± 0.02 nm, respectively. The calculated minimum diameter is comparable to that of the molecular cross section of DBNO (0.53 × 0.70 × 0.45 nm^3^, see 1. Introduction) as well as that of the TPP/benzene IC calculated on the basis of reference 3; 0.52 and 0.79 nm.

**Figure 2:**
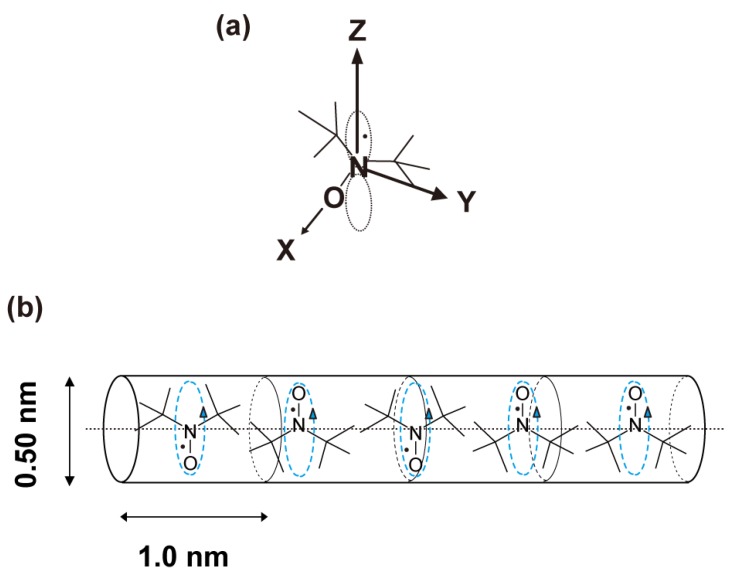
Molecular axes of DBNO **(a)** and the packing model of DBNO molecules in the TPP nanochannels **(b)**. According to the composition of **1** determined by CA, TG, and spin concentration measured by ESR, it is expected that 1.25 DBNO molecules are accommodated in the TPP unit cell formed by two TPP molecules, *i.e.*, five DBNO molecules are included in four TPP unit cells. In Figure 2(b), all DBNO molecules are depicted only in the upward or downward form for reasons of expediency, although in fact, the orientation of their nitroxide groups are expected to be disordered in a vertical plane to the channel axis of the TPP nanochannels. The cyan dashed curly arrows represent the uni-axial rotation of DBNO molecules around the channel axis.

On the basis of the diameter dimensions of the TPP nanochannel moiety of **1,** the molecular orientation of DBNO is suggested as follows: if the molecular axes are taken as Figure 2(a), DBNO in the TPP nanochannels is oriented in the molecular Y axis to be parallel to the channel axis of the nanochannels (Figure 2b), as would be expected in the case of TPP/TEMPO or [TPP/(TEMPOL)_0.5_-(mesitylene)_0.25_] IC prepared by the co-precipitation method (see I. Introduction). In other words, this means that the nitroxide group of DBNO is orientated in a plane that is vertical to the channel axis. In Figure 2(b), all DBNO molecules are depicted only in the upward or downward form for reasons of expediency. In fact, the orientation of their nitroxide group is expected to be disordered in the plane. Therefore, in Figure 1(b), the XRD patterns based on the periodical arrangement of the guest compounds are unexpected. Compared with the dimension of the TPP unit cell of **1,** based on the above estimation, it is found that DBNO molecules can be included even in the minimum diameter moiety of the TPP nanochannels.

In addition, the molecular orientation of DBNO in the TPP nanochannels can be anticipated also on the basis of the composition of **1**. Since the channel axis of the TPP nanochannels corresponds to the *c*-axis of the TPP hexagonal lattice, the dimension of the channel direction in a TPP unit cell is equal to the cell parameter *c*. In **1**, *i.e.*, TPP/(DBNO)_0.62_ IC, 1.24 DBNO molecules are expected to be accommodated per unit cell formed by two TPP molecules. In other word, it is believed that five DBNO molecules can be accommodated in four TPP unit cells as shown in Figure 2(b). This means that the channel direction dimension of the TPP nanochannels occupied by one DBNO molecule is estimated to be about 0.8 nm, because the estimated cell parameter *c* of **1** is about 1 nm. As the dimension of a DBNO molecule along the molecular Y axis is 0.70 nm, the above molecular orientation is consistent with the experimental results. In most of the TPP inclusion compound, the number of guest molecules per TPP unit cell is not unity, and it is common that the periodicity of (the center of gravity of) the guest compound in the TPP nanochannels is not coincident with that of the host structure [2]. This implies that the wall-guest interaction is very weak. This may be because the wall is formed by the phenyl rings of the TPP molecules, which are *π*-electron-rich.

In the case of TPP/TEMPO or [TPP/(TEMPOL)_0.5_-(mesitylene)_0.25_] IC prepared by the co-precipitation method, it was suggested that the center of gravity of organic radicals are located in the maximum diameter part of the TPP nanochannels [32] because the maximum diameter is almost the same as the dimension between the nitroxide group and the 4-position substitution group of TEMPO or TEMPOL. On the other hand, the determination of the position of (the center of gravity of) DBNO molecules along the TPP channel axis is difficult, because of the different periodicity of guest molecules in the TPP nanochannels to the host structure. In addition, it is suggested that DBNO molecules are uni-axially rotating around the channel axis of the TPP nanochannels as expected in most of the TPP IC, because of the low friction between the guest molecules and the wall of the nanochannels (the cyan dashed curly arrows in Figure 2(b) shows the uni-axial molecular rotation of DBNO). For the determination of the detailed structure, the preparation of the single-crystal and the measurements of single-crystal XRD of **1** are now underway.

### 2.2. ESR measurements of compound **1** *(TPP/(DBNO)_0.62_ IC)*

**Figure 3:**
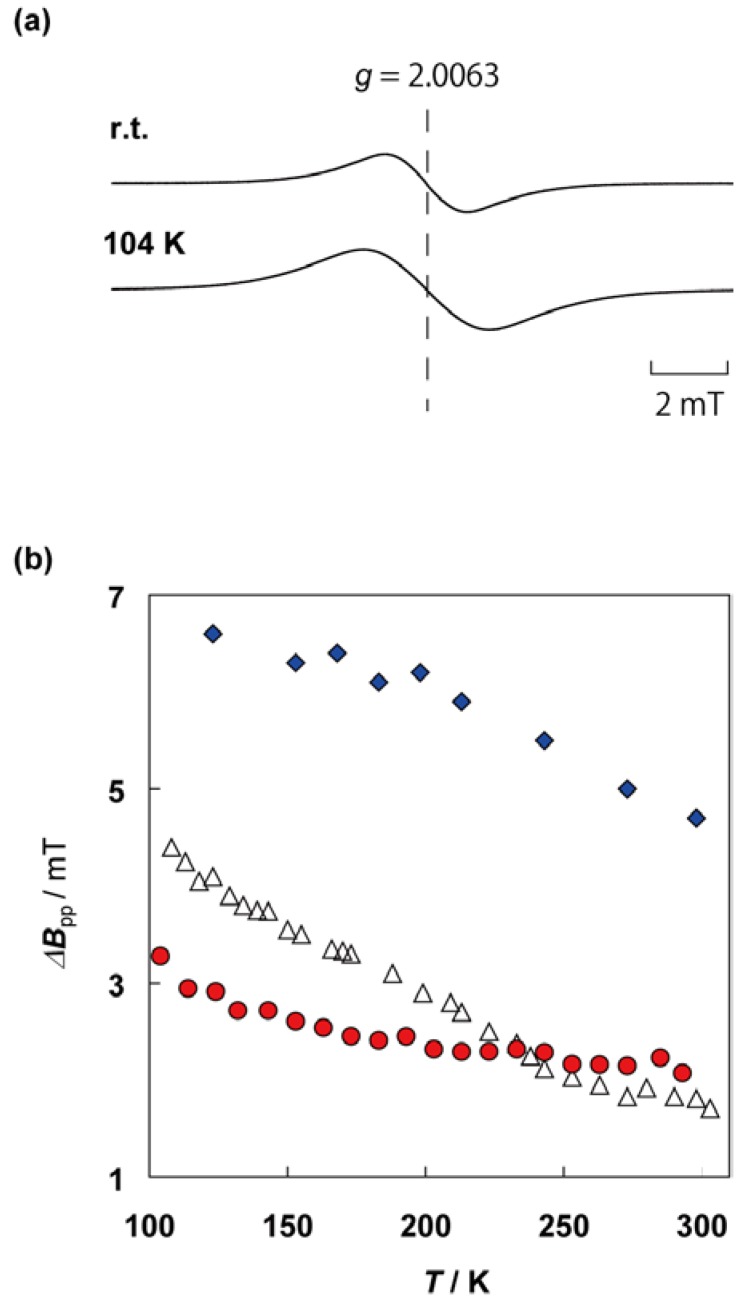
Powder ESR spectra of compound **1** measured at 293 K (top) and 104 K (bottom) **(a)**, and temperature dependence of the peak-to-peak line width, *ΔB*_pp_, of **1** (red circles), TPP/TEMPO IC (open triangles) and [TPP/(TEMPOL)_0.5_-(mesitylene)_0.25_] IC (blue diamonds), respectively **(b)** [25,32].

The measurement of the ESR spectra of **1** was carried out from 104 to 293 K. In the whole temperature range, the ESR spectra were isotropic as shown in Figure 3(a). Figure 3(b) shows temperature dependence of the peak-to-peak line width, *ΔB*_pp,_ of **1** (red circles) from 104 to 293 K, as well as that of TPP/TEMPO IC (open triangles) [25] and [TPP/(TEMPOL)_0.5_-(mesitylene)_0.25_] IC prepared by the co-precipitation method (blue diamonds) [32]. *ΔB*_pp_ of **1** slightly narrowed from 3.3 mT at 104 K to 2.1 mT at 293 K with increasing temperature. Above 230 K, *ΔB*_pp_ of **1** was slightly larger than that of TPP/TEMPO IC. However, the broadening of the ESR line of **1** was much more gradual compared with that of TPP/TEMPO IC when the temperature is lowered. The difference of temperature dependence of the line width between **1** and TPP/TEMPO IC is expected to be caused by the balance between the intrinsic line width due to dipolar interaction and the magnitude of the line-narrowing due to inter-spin exchange interactions and/or motional averaging. As the intra-chain inter-spin distances are shorter in **1** than TPP/TEMPO IC, according to the discussion in section 2.1, it is straightforward to expect that the dipolar line broadening is larger in **1** than TPP/TEMPO IC. On the other hand, the line narrowing of **1** and TPP/TEMPO IC is closely related to their molecular dynamics in the TPP nanochannels. The relatively large molecular size of TEMPO interrupts the free molecular reorientation in the TPP nanochannels at low temperatures [26]. Meanwhile, DBNO molecules are anticipated to uni-axially rotate in the TPP nanochannels even at 104 K with adequate speed to induce the line narrowing, because their smaller molecular cross-section does not interrupt the molecular rotation in the TPP nanochannels. This is consistent with the fact that many medium-sized guest molecules with a similar molecular cross section to DBNO, such as trioxane, perform rotational motion in the TPP nanochannels even at 90 K [20]. It should be noted that despite the spin concentration of **1** (6.8 × 10^20^ g^-1^) being 1.2 times higher than that of TPP/TEMPO IC (5.6 × 10^20^ g^-1^), the line width at room temperature is comparable. According to these considerations, **1** is expected to show stronger line-narrowing than TPP/TEMPO IC. For clarification of the temperature dependence of the line-width, and of the line-narrowing mechanism of **1**, ESR measurements below 77 K are now underway.

In addition, the isotropic ESR spectra of **1** from 104 K to 293 K were approximated by a superposition of Lorentzian and Gaussian functions. Above 150 K, the ratio of Lorentzian and Gaussian components is almost constant at 64:36. Below 150 K, the ratio of Gaussian components slightly increased, and finally, it reached up to 46% at 104 K. It is expected that the ratio of Gaussian components in the ESR spectra monotonically increases with decreasing temperature as observed for TPP/TEMPO IC, since the line-narrowing due to motional averaging becomes weak at low temperatures [25]. In general, the ESR line shape is determined by the Fourier transform of the relaxation function of transverse magnetization, *φ*(*t*) [36]. *φ*(*t*) is expressed as follows:

(1) $$\phi (t)=\exp[-M_{2}\int_{0}^{t}(t-\tau )\Psi (\tau )d\tau ]$$

where *M*_2_ is the second moment of the resonance line, and *Ψ*(*τ*) the normalized transverse magnetization correlation function of the local field at the spin. If *Ψ*(*τ*) decays according to the diffusion equation at the long time region ( $\tau_{e}<<\tau$) characterized by $\tau_{e}$ ~ $1/\omega_{e}$ ( $\omega_{e}$ the spin fluctuation frequency), $\Psi (\tau )\propto \tau^{-d/2}$(*d* the dimensionality of the system). Then, in the 1-D spin diffusion process, *φ*(*t*) is represented by exp[-(*Γt*)^3/2^] using a constant *Γ*, and it is suggested that exchange interaction exists between the neighboring spins in the 1-D system. In this process, since the effect of exchange narrowing becomes weaker than the case of three-dimensional exchange interaction between the spins, the ESR line shape becomes intermediate between Lorentzian and Gaussian.

**Figure 4:**
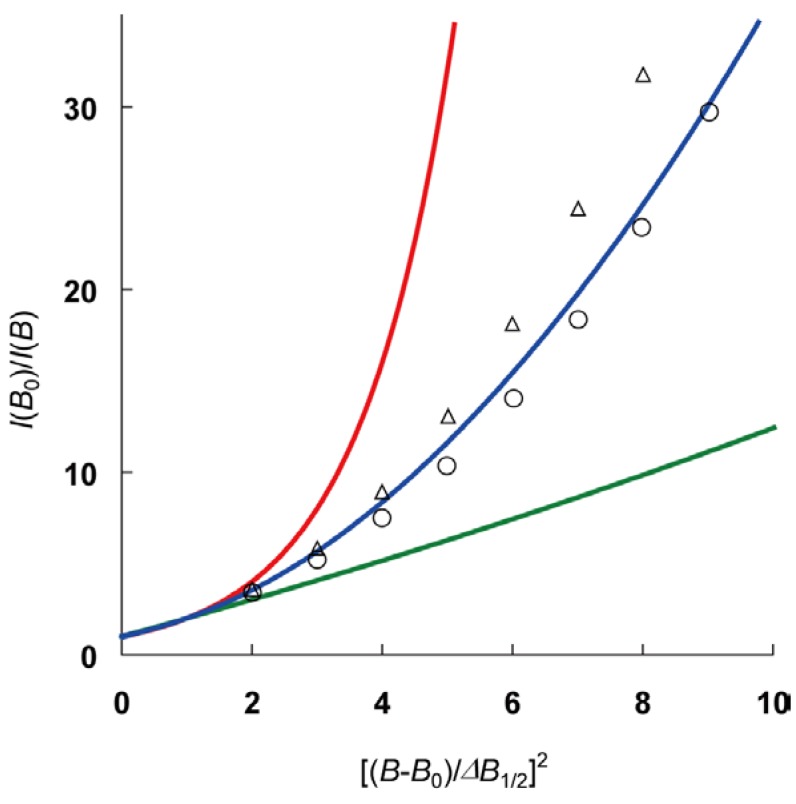
Dietz’s plot [25,36] of the line shape of X-band ESR spectra of compound **1** at 293 K (circles) and 104 K (triangles). Colored curves show the line shape and the inter-spin behavior: a Lorentzian (green, 3-D exchange interaction or motional narrowing), a Gaussian (red, only dipolar interaction), and an intermediate between them (blue, 1-D spin diffusion).

Figure 4 shows the Dietz’s plot of the ESR line shape of **1** at 104 and 293 K for the analysis of the dimensionality of inter-spin interaction [25,36]. In this plot, the colored curves show the line shape of the isotropic spectrum with different inter-spin interaction: a Lorentzian (green, 3-D exchange interaction or motional narrowing), a Gaussian (red, only dipolar interaction), and an intermediate between them (blue, 1-D spin diffusion). At a glance, the experimental results at 293 K coincide well with the curve showing 1-D spin diffusion, whereas at 104 K, the experimental results slightly approach the Gaussian curve. The fact that the ESR line shape of **1** is not pure Lorentzian, but intermediate between Lorentzian and Gaussian, suggests that the averaging by the molecular rotation of DBNO in the TPP nanochannels is inadequately achieved, and that the inter-spin interaction in this system can be explained using the 1-D spin diffusion model. Since it is anticipated that the composition of compound **1** is homogeneous and the DBNO molecules are rotating in the nanochannel (see above), it is unexpected that two different kinds of ESR detectable specimens showing Lorentzian and Gaussian, respectively, are observed by chance. As a result, it is strongly suggested that the inter-spin interaction in **1** is governed by the 1-D spin diffusion process.

In summary, these results imply that **1**, *i.e.*, TPP/(DBNO)_0.62_ IC, is a remarkable organic material with 1-D spin chain and showing 1-D spin diffusion at room temperature. In order to clarify the bulk magnetism and elucidate the type of the intra-chain exchange interaction of TPP/(DBNO)_0.62_ IC, the low-temperature magnetic susceptibility measurements are now underway as well as ESR measurements below 77 K.

### 2.3. Sample characterization and evaluation of compound **2**

**Figure 5:**
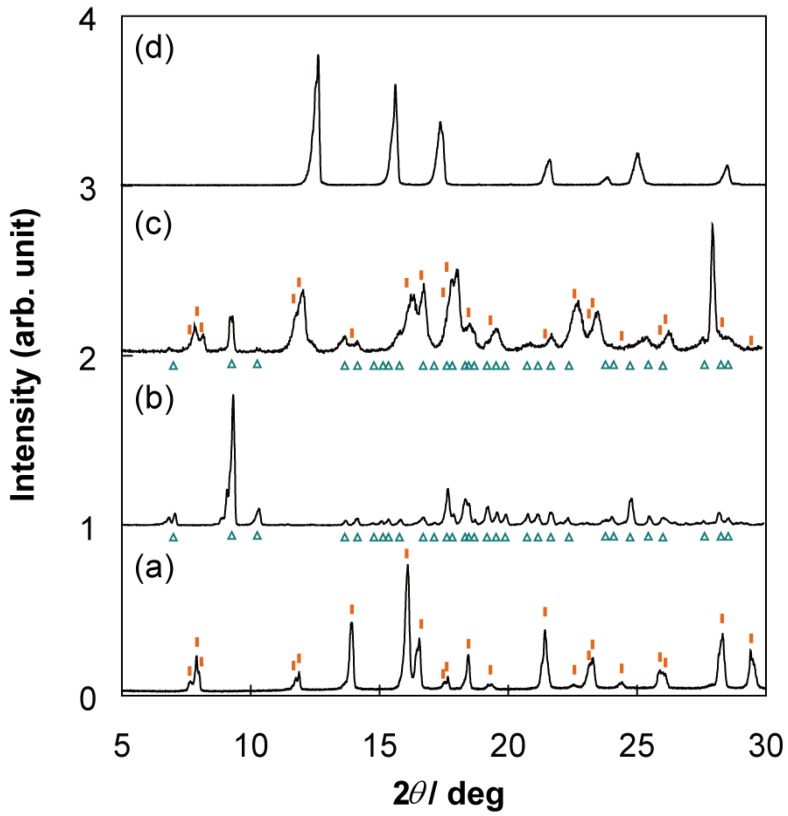
Powder X-ray pattern of [TPP/(TEMPOL)_0.5_-(mesitylene)_0.25_] IC prepared by the co-precipitation method [32] **(a)**, TPP monoclinic phase recrystallized from mesitylene **(b)**, compound **2 (c)**, and bulk TEMPOL **(d)**. The typical peak positions of **(a)** or **(b)** were depicted with red vertical bars on **(a)** or cyan triangles under **(b)**, respectively. The powder pattern of **(c)** was almost interpreted as a superposition of that of **(a)** and **(b)**. The peaks corresponding to the bulk TEMPOL in **(d)** were hardly observed in **(c)**.

Chemical analysis of **2** revealed the following: H, 3.87%; C, 52.64%; N, 8.13%. The results closely approximated the composition of TPP:TEMPOL:mesitylene = 1:0.23:0.46 within experimental error: H, 3.94%; C, 52.48%; N, 8.16%. These results obviously imply co-inclusion of mesitylene. The molar ratio of TEMPOL to TPP is two-times smaller in **2** than [TPP/(TEMPOL)_0.5_-(mesitylene)_0.25_] IC prepared by the co-precipitation method [32], whereas that of mesitylene is two-times larger. These results were confirmed by TG: the weight ratio of the compounds desorbed from **2** was 16.6%, while the estimation in TPP:TEMPOL:mesitylene = 1:0.23:0.46 was 17.0%. Moreover, the ESR spin concentration of **2**, 3.0 × 10^20^ g^-1^, was well coincident with the expected value of 2.5 × 10^20^ g^-1^ for TPP:TEMPOL:mesitylene = 1:0.23:0.46. Figure 5 shows powder X-ray patterns of [TPP/(TEMPOL)_0.5_-(mesitylene)_0.25_] IC prepared by the co-precipitation method [32], TPP monoclinic phase in which no guest molecule can be included [4], **2**, and the bulk TEMPOL. Although the powder pattern of **2** (Figure 5c) is complicated, this can be interpreted as a superposition of that of Figure 5(a) and (b); the peak positions in Figure 5(c) correspond to the ones depicted by red bars on Figure 5(a) or cyan triangles under Figure 5(b). The peaks corresponding to the bulk TEMPOL in Figure 5(d) was hardly observed in Figure 5(c). The existence of TPP monoclinic phase is consistent with the relatively larger ratio of TPP in **2** than [TPP/(TEMPOL)_0.5_-(mesitylene)_0.25_] IC prepared by the co-precipitation method. These results imply that 1-D TPP nanochannels in **2** include TEMPOL only partially.

**Figure 6:**
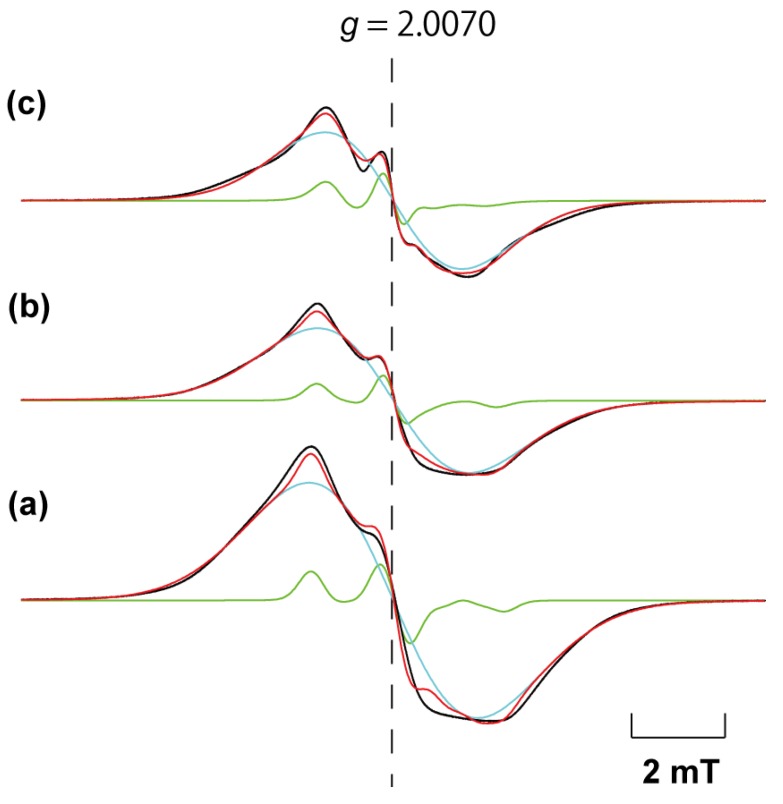
Powder ESR spectra of compound **2** at 143 K **(a)**, 223 K **(b)**, and 283 K **(c)**. All spectra (black line) were reproduced by a superposition (red line) of two temperature-dependent components: an isotropic broad signal (cyan) and an anisotropic slow averaging one (green), as observed in the TPP/TEMPO IC or the IC using TEMPOL and TPP prepared by the adsorption method [24,32].

Temperature dependence of the ESR spectra of **2** from 103 K to 293 K was investigated. Figure 6 shows the ESR spectra of **2** at 103 K, 223 K, and 285 K. All spectra (black line) were reproduced by a superposition (red line) of two temperature-dependent components: an isotropic broad signal (cyan) and an anisotropic slow averaging one (green), as observed in the TPP/TEMPO IC or the IC using TPP and TEMPOL prepared by the adsorption method [24,32]. In these cases, each component is originated from two different physical states: a densely-packed organic radical part in the TPP nanochannels and an isolated radical part in the nanochannels. In the TPP/TEMPO IC, molecular orientation and dynamics could be determined [26]. Since molecular rotational motion of the radical in the TPP nanochannels is relatively slow, the averaging of the ESR spectrum is incompletely achieved. These cause the ESR spectrum to make it complicated [27]. On the basis of the above characterization and the process of sample preparation (see section 3.2), for compound **2** it is expected that at least two different kinds of ESR detectable specimens are produced: a densely-packed TEMPOL (co-included by mesitylene) and an isolated TEMPOL molecule. The former can be constructed by using similar mechanism as reported in our previous work [32] of [TPP/(TEMPOL)_0.5_-(mesitylene)_0.25_] IC prepared by the co-precipitation method, whereas the latter is expected to be produced by the circumstance where TEMPOL molecules are separated by excess co-included mesitylene molecules.

In the preparation process of **2**, powder TEMPOL was excessively added to make a numerical advantage condition for the preferential inclusion of TEMPOL in the TPP nanochannels. However, only a small amount of TEMPOL can be dissolved in mesitylene and most of TEMPOL molecules aggregate because of their strong intermolecular force, such as hydrogen bonding. As a result, mesitylene molecules are co-included in the TPP nanochannels, and a small amount of TEMPOL molecules dissolved in mesitylene are isolated from each other by apparent excess mesitylene molecules. This implies that it is difficult for TEMPOL to be included in the nanochannels without co-inclusion of mesitylene in the co-precipitation or recrystallization process. Therefore, the isolated TEMPOL part of **2** may be to some degree the same as the IC using TPP and TEMPOL prepared by the adsorption method in which TEMPOL molecules exist intermittently in the nanochannels.

In Figure 6, the ESR spectrum was independent on temperature below 143 K. All spectra were reproduced by a superposition of (95 ± 1)% isotropic component and (5 ± 1)% slow averaging component, respectively, and the population of the two components was independent of temperature. Slow averaging component was reproduced by the similar molecular orientation and dynamics as observed in TPP/TEMPO IC and [TPP/(TEMPOL)_0.5_-(mesitylene)_0.25_] IC: the nitroxide group of the radical molecule is orientated perpendicular to the channel axis and they uni-axially rotate in the TPP nanochannels around the channel axis [26,32]. At 143 K, the spectrum was reproduced by a superposition of a rigid-limit powder spectrum of TEMPOL corresponding to the isolated TEMPOL molecules in the TPP nanochannels and the isotropic line with *ΔB*_pp_ = 5.8 mT and Gaussian:Lorentzian = 91:9 corresponding to the densely-packed TEMPOL part. The principal values of ***g*** and ***A*** tensors are given in reference 32. The line shape of the isolated TEMPOL component was averaged with increasing temperature, and a correlation time of rotational motion, *τ*_R_, was estimated to be 9 × 10^-8^ s at 285 K. This is comparable to *τ*_R_ = 5 × 10^-7^ s at 293 K of the IC using TPP and TEMPOL prepared by the adsorption method [32]. This implies that co-inclusion of mesitylene hardly contributes to molecular motion of TEMPOL in the TPP nanochannels. The line width of the isotropic component was slightly narrowed from 5.8 mT at 143 K to 4.8 mT at 283 K and the weight ratio of Lorentzian component slightly increased. Temperature dependence of the isotropic component is comparable with that of [TPP/(TEMPOL)_0.5_-(mesitylene)_0.25_] IC prepared by the co-precipitation method [32]. According to these results, the powder XRD pattern depicted by the red bars in Figure 5(c) is expected to originate not only from [TPP/(TEMPOL)_0.5_-(mesitylene)_0.25_] IC but also the IC of TPP and TEMPOL diluted by mesitylene.

According to these results, we should rethink the preparation method for pure TPP/TEMPOL IC. In order to prepare the inclusion compound using TPP and TEMPO derivatives in which the size of guest molecules is larger than TEMPO, we are currently planning a new inclusion compound using TPP and 4-oxo-TEMPO (TEMPONE), because it has not so high polarity as TEMPOL. Further works are now underway.

## 3. Experimental Section

### 3.1. Chemicals

Tris(*o*-phenylenedioxy)cyclotriphosphazene (TPP) was synthesized as described in the literature [24]. The synthesized specimen was recrystallized twice from benzene for purification. A guest-free TPP powder specimen was prepared by heating at 348 K for three hours under reduced pressure. Di-*t*-butyl nitroxide were purchased from Sigma-Aldrich Japan K.K. 4-Hydroxy-2,2,6,6-tetramethyl-1-piperidinyloxyl (TEMPOL) and 4-methoxy-2,2,6,6-tetramethyl-1-piperidinyloxyl (MeO-TEMPO) radical, and 1,3,5-trimethylbenzene (mesitylene) were purchased from Tokyo Chemical Industry Co. Ltd. (TCI). All chemicals were used without further purification.

### 3.2. Sample preparation

Compound **1** (TPP/(DBNO)_0.62_ IC) was prepared by the following procedure: Liquid DBNO (1 g) was dissolved in a saturated mesitylene solution of TPP (2 mL) at 273 K. Light-orange powder specimens were co-precipitated after the resultant solution was kept cool under ambient pressure for an hour. The crystals were filtered and dried in air without washing to avoid the solvation of the inclusion compound. The prepared IC was stable for at least half a year under standard conditions.

The IC using TPP and TEMPOL (compound **2**) was prepared according to the following procedure: A large excess of powdered TEMPOL (2 g) was dissolved in 2 mL of a saturated mesitylene solution of TPP at 333 K. The resultant solution was cooled at room temperature immediately to prevent thermal decomposition of TEMPOL; pink powder specimens and excess TEMPOL crystals were co-precipitated after several hours. The crystals were filtered, washed with water several times to remove excess TEMPOL crystal, and dried in air. The prepared IC was stable for at least several months under standard conditions.

It was found that 1,3,5-triethylbenzene, which we suggested in our previous study [32] as one of proper solvents to prepare “TPP/TEMPOL IC”, was not suitable because of the quite low TEMPOL solubility in the solvent. TEMPOL molecules have a great tendency to aggregate in nonpolar solvents because of strong intermolecular forces, such as hydrogen bonding. On the other hand, the use of polar solvents for the preparation of “TPP/TEMPOL IC” is also inadequate because TPP is poorly soluble in them. In addition, we attempted the preparation of TPP/TEMPOL IC using 1,3,5-trimethoxybenzene as a solvent, which is larger than TEMPOL or mesitylene, and which can dissolve both of them. However, no specimen was obtained when the solution was cooled. In the preparation of pure TPP/TEMPOL IC, we should intrinsically rethink the preparation method.

The preparation of the IC using TPP and MeO-TEMPO was performed according to the following procedure: Gas adsorption of MeO-TEMPO (melting point 313 K) was carried out according to our previous study [24]. Despite one-day exposure, the color of TPP crystal was unchanged and any weight increase was not observed. Meanwhile, in the recrystallization process, although an excess of powdered MeO-TEMPO (1 g) was dissolved in a saturated mesitylene solution of TPP (2 mL) at 333 K, no crystal was co-precipitated. According to the results, it was concluded that the inclusion of MeO-TEMPO in the TPP nanochannels was interrupted because of the large molecular size (0.98 × 0.87 × 0.64 nm^3^).

Sample characterization of **1** and **2** was conducted using chemical analysis (CA), powder X-ray diffraction (XRD), thermogravimetric analysis (TG) and ESR spin concentration measurements. The assignment using solution ^1^H or ^13^C NMR measurements was difficult because of the decomposition of DBNO in heating for dissolution or the extreme line broadening by the strong relaxation effect based on the unpaired spin of DBNO.

### 3.3. Instruments

Powder XRD analyses for all samples were carried out using a diffractometer (Rint2100; Rigaku Corp.) with graphite monochromated Cu-Kα radiation (*λ* = 0.15418 nm) at room temperature. Data were collected in the *θ*–2*θ* scan mode using a 2*θ* scan rate of 1° min^-1^; the 2*θ* collection range was 3–90°.

Powder ESR spectra were recorded using an X-band spectrometer (JES-FA200; JEOL) at temperatures of 104 K to room temperature. Powdered specimens of 2–3 mg were packed in an ESR tube (270 mm long, o.d. 5 mmφ) made from quartz glass, then capped using a Teflon seal in a dried-air atmosphere. Thermal equilibrium of the sample was achieved by waiting 5–10 min after temperature changes. We also confirmed signal reproduction in both directions of increasing and decreasing temperature. The X-band microwave power was set to 0.01, 1, and 10 mW under non-saturated conditions. Spectral simulations of compound **2** were performed using the Chili program software package (EasySpin 3.1.1; ETH Zürich) [37].

## 4. Conclusions

According to a sequence of sample preparations, it was found that a guest organic radical with relatively small molecular size, such as DBNO, can easily be included in TPP nanochannels, and that such ICs enable 1-D spin chains. The success of the preparation of organic ICs with 1-D spin chains other than TPP/TEMPO IC is evident as a possibility to develop new 1-D organic magnets [38]. On the other hand, it was concluded that the inclusion of organic radicals with high polarity, such as TEMPOL, is difficult without co-inclusion of solvent molecules. In the preparation of the IC using TPP and organic radical, the suitable combination of guest compound and solvent should be selected in view of not only molecular size of radical molecules but also polarity. We believe that these results can shed new light on the design of a new organic magnet. Further works, such as ESR measurements below 77 K and magnetic susceptibility measurements using SQUID magnetometer, are now underway.
